# Neuroprotective effects of fosgonimeton and amyloid‐β‐targeting monoclonal antibodies against amyloid‐β toxicity in primary neuron culture

**DOI:** 10.1002/alz.089826

**Published:** 2025-01-09

**Authors:** Sherif M Reda, Wei Wu, Sharay E. Setti, Andrée‐Anne Berthiaume, Robert W Taylor, Jewel L Johnston, Kevin J. Church

**Affiliations:** ^1^ Athira Pharma, Inc., Bothell, WA USA

## Abstract

**Background:**

We have previously reported the neuroprotective effects of fosgonimeton in amyloid‐β (Aβ)‐driven preclinical models of Alzheimer’s disease (AD). Fosgonimeton is an investigational small‐molecule positive modulator of the neurotrophic hepatocyte growth factor (HGF) system, currently under investigation for mild‐to‐moderate AD (LIFT‐AD; NCT04488419). Given the recent approvals of Aβ‐targeting monoclonal antibodies (Aβ‐mAbs) for the treatment of AD, and growing recognition that combination therapies may improve treatment outcomes, we sought to investigate the preclinical activity of fosgonimeton in the presence of Aβ‐mAbs. Here, we report the neuroprotective effects of fosgonimeton against Aβ toxicity in primary cortical neurons in the presence and absence of lecanemab, an Aβ‐targeting mAb that has received full approval for early AD.

**Method:**

Primary rat cortical neurons were pre‐treated with the active metabolite of fosgonimeton (fosgo‐AM; 100 nM), lecanemab (25 nM), or fosgo‐AM + lecanemab for 15 minutes, and subsequently challenged with Aβ1‐42 (15 µM). In another condition, the drug treatments were co‐applied with Aβ1‐42. Following 24‐ or 48‐hour Aβ exposure, cultures were immunoassayed for microtubule‐associated protein‐2 (neuronal marker) to determine neuronal survival and neurite network length.

**Result:**

Cortical neurons pre‐treated with fosgo‐AM and/or lecanemab exhibited a significant increase in neuronal survival and neurite network length following 24h or 48h Aβ challenge. In the Aβ1‐42 co‐application conditions, following 24h Aβ exposure, all treatments significantly promoted neuronal survival, but only the combination of fosgo‐AM + lecanemab significantly preserved neurite networks. In the Aβ1‐42 co‐application conditions, following 48h Aβ exposure, only the combination of fosgo‐AM + lecanemab significantly promoted neuronal survival, and none of the treatments had a significant effect on neurite network.

**Conclusion:**

This study provides preliminary evidence that, on a cellular level, the neuroprotective ability of fosgonimeton against Aβ‐mediated toxicity in primary neurons is maintained in the presence of lecanemab. Furthermore, in some conditions, only the combination of fosgo‐AM and lecanemab demonstrated a significant increase in neuronal survival. While additional experiments are required to characterize these effects, these initial observations may have important implications regarding the potential of fosgonimeton as a treatment for AD, with or without Aβ‐targeting antibodies.

